# Empowerment ability of main caregivers of children with intracranial tumors after surgery: a cross-sectional survey

**DOI:** 10.3389/fped.2025.1682115

**Published:** 2025-11-13

**Authors:** Wen Zhou, Wen Yang, Tian Cao

**Affiliations:** Department of Neurosurgery, Children’s Hospital of Nanjing Medical University, Nanjing, Jiangsu, China

**Keywords:** empowerment, caregiver, children, pediatric, neurosurgery, nursing, care

## Abstract

**Background:**

The empowerment capacity of caregivers exerts a significant impact on children's prognosis. This study aims to analyze the empowerment ability of primary caregivers of children who have undergone craniotomy, thereby providing evidence-based support for pediatric nursing practice.

**Methods:**

Participants in this study included main caregivers of children with intracranial tumors who underwent surgical treatment in our hospital between February 2022 and April 2025. The empowerment ability of these main caregivers was assessed using the Main Caregivers’ Empowerment Measurement (MCEM) scale. Correlation analysis and multiple linear stepwise regression analysis were performed to identify potential influencing factors.

**Results:**

A total of 242 main caregivers were enrolled in this study. The average empowerment score among these caregivers was 160.64 ± 15.07. The “Personal Resources” and “Care Knowledge and Skills” dimensions yield the lowest scores across all dimensions. Correlation analysis demonstrated that the child's age (r = 0.581), place of residence (r = 0.546), caregiver's gender (r = 0.604), caregiver's age (r = 0.626), educational level (r = 0.615), and monthly per capita income (r = 0.586) were all significantly associated with the empowerment ability (all *p* < 0.05). Furthermore, multivariate linear regression analysis identified those factors as significant influencing factors of caregiver empowerment. These selected variables accounted for 58.8% of the variance in caregivers’empowerment ability.

**Conclusion:**

Given the substantial room for improvement in the empowerment levels of main caregivers, targeted strategies are urgently needed to address this gap. Healthcare professionals should therefore design interventions that are tailored to the identified influencing factors to enhance caregivers’ empowerment capacities.

## Introduction

Intracranial tumors are among the most prevalent solid tumors in childhood, ranking second in pediatric malignancy incidence with a rising trend in recent years (1). Owing to the immature pediatric nervous system, tumor location, invasiveness, and treatment procedures frequently induce complications such as neurological dysfunction, cognitive delay, and motor limitations (2, 3), necessitating long-term or lifelong family care. Compared to adult patients, pediatric intracranial tumor care demands are more specialized—encompassing basic daily support (e.g., diet, routines) alongside postoperative rehabilitation, psychological interventions, and treatment adherence management (4).This imposes substantial physical, psychological, and economic burdens on primary caregivers (predominantly parents), whose care capacity directly impacts treatment outcomes, rehabilitation progress, and quality of life (5). Notably, current clinical interventions prioritize the child's disease over systematic caregiver support, leaving some caregivers in a “passive care” predicament without professional guidance (6).

Empowerment, a cornerstone of health promotion, enhances individuals' knowledge, skills, and initiative to engage in decision-making and assume responsibilities (7). In pediatric chronic disease care, caregiver empowerment is defined as “the comprehensive ability to recognize care roles, acquire resources, participate in medical decisions, and resolve care-related issues” (8), with its level closely linked to care quality, child health outcomes, and caregiver well-being. Studies (9, 10) confirm that highly empowered caregivers better manage care-related stress, reduce negative emotions, and collaborate more effectively with medical teams. However, research gaps persist regarding postoperative intracranial tumor caregivers: their empowerment status remains understudied, with insufficient data on care knowledge, decision-making participation, and resource utilization; furthermore, key influencing factors (e.g., medical environment, social support, caregiver characteristics) lack systematic conclusions, hindering targeted interventions.

While family-centered care models advance in pediatrics, improving caregiver empowerment has become critical for optimizing pediatric oncology care. Although empowerment interventions for caregivers of children with other chronic diseases (e.g., leukemia, cerebral palsy) have proven effective via personalized training, shared decision-making, and social resource integration, the unique context of pediatric intracranial tumors—long rehabilitation cycles, high complication risks, and the need for integrated family-medical care—renders existing models inapplicable. Thus, there is an urgent need to develop tailored nursing strategies based on this population's empowerment status. This study aims to evaluate the empowerment of primary caregivers of children post-intracranial tumor surgery, addressing research gaps to inform precise clinical nursing plans, foster a “medical-family” collaborative model, improve children's long-term health outcomes, and alleviate caregiver burdens.

## Methods

### Study design

This study employed a cross-sectional survey design, which involves one-time data collection to objectively characterize the current empowerment levels of primary caregivers of children post-intracranial tumor surgery and identify associated influencing factors.

### Ethical consideration

The research protocol was rigorously reviewed and approved by the Institutional Medical Ethics Committee (approval number: 202509004-1), with the entire study conducted in strict compliance with the human research ethical guidelines outlined in the Declaration of Helsinki to fully safeguard participants' rights and interests. All participants provided written informed consent after comprehensively understanding the study's purpose, content, potential risks, and data confidentiality measures. Trained researchers guided this process to ensure voluntary participation without coercion. Participants were also explicitly informed of their right to withdraw from the study at any time, with no impact on their access to standard medical services.

### Study population

The research focused on primary caregivers of children diagnosed with intracranial tumors. These children underwent surgical treatment in the Neurosurgery Department of this hospital. The recruitment period spanned from February 2022 to April 2025. The inclusion criteria were strictly defined as follows: ① Children were clearly diagnosed with intracranial tumors by cranial computed tomography (CT) or magnetic resonance imaging (MRI) and underwent tumor resection surgery in this hospital; ② Caregivers were aged ≥ 18 years, had a blood relationship with the child (such as parents, grandparents, etc.) or a legally recognized guardianship relationship (such as legal guardians, etc.), and were able to assume the main care responsibilities; ③ The duration of continuous care for the child was ≥1 month, and the average daily care time was ≥4 h, ensuring that they had sufficient experience in the postoperative care process of the child; ④ Caregivers were conscious, had basic language comprehension and expression abilities, could independently complete the questionnaire, and voluntarily cooperated with the data collection work of this study.

During the research process, individuals with the following situations were excluded: caregivers who clearly expressed their unwillingness to participate in this survey or refused to sign the informed consent form; caregivers who were unable to communicate effectively or complete the questionnaire due to mental disorders, cognitive dysfunction, or other reasons.

### Survey tools

The following relevant information of main caregivers was collected and surveyed: the child's gender, the child's age, place of residence, medical expense payment method, caregiver's gender, caregiver's age, relationship with the child, educational level, employment status, monthly per capita income, presence of chronic diseases, religious belief, average daily hours spent caring for the child, and presence of other caregivers for the child.

In this study, the Main Caregivers' Empowerment Measurement (MCEM), developed and validated by Wu et al. (11), was adopted as the core measurement tool to assess the empowerment level of main caregivers of children with intracranial tumors after surgery. This scale has undergone rigorous development procedures and reliability and validity tests, demonstrating good applicability and scientificity. Derived from the multidimensional roles and practical scenarios of caregivers, the MCEM scale consists of 9 dimensions: “Relationship with the Care Recipient”, “Benevolent Care”, “Expectations for Care Outcomes”, “Caregiver's Subjectivity”, “Care Beliefs”, “Perception of Care's Role”, “Personal Resources”, “Concerns About the Surroundings”, and “Care Knowledge and Skills”. These 9 dimensions are interrelated yet each has its focus, comprehensively covering core elements of caregivers' empowerment process, such as emotional connection, care motivation, self-cognition, resource utilization, and skill mastery. The scale comprises a total of 51 items, using a 4-point Likert scoring method, where 1 point represents “Not at all”, 2 points represent “Slightly not”, 3 points represent “Slightly yes”, and 4 points represent “Usually”. Higher total scores and subscale scores indicate stronger empowerment ability of caregivers, reflecting better performance in terms of initiative, resource integration ability, awareness of decision-making participation, and problem-solving effectiveness during the care process. Reliability test results showed that the Cronbach's α coefficient of the Chinese version of the MCEM scale was 0.89, indicating good overall internal consistency of the scale (12). The measurement results are highly reliable and stable, which can provide solid data support for the quantitative assessment of caregivers' empowerment ability in this study.

### Data collection

The data collection process of this study strictly followed a standardized protocol, with specific implementation procedures as follows: First, researchers introduced themselves to the main caregivers of children with intracranial tumors, and elaborated on the research purpose, core content, academic significance, and data usage of this survey. Written informed consent was obtained on the premise that caregivers fully understood the study and voluntarily agreed to participate. Subsequently, paper questionnaires were distributed to eligible caregivers, who were instructed to complete the questionnaires independently on-site.

For caregivers facing difficulties in filling out the questionnaires due to reading barriers, limited educational level, or emotional distress, researchers who had received unified training read out the content of each item using standardized instructions. They provided standardized answers to the caregivers' questions to ensure that the latter accurately understood the meaning of each item. The researchers then filled in the questionnaires on behalf of the caregivers strictly according to their true expressions, avoiding any suggestive hints throughout the process to ensure the objectivity of the data.

After the questionnaires were completed, researchers collected and conducted a preliminary review on-site. For parts with missing information, repeated options, or logical contradictions, they immediately communicated and verified with the caregivers, guiding them to supplement or correct the relevant content. Clear criteria were followed for excluding invalid questionnaires: those with a large number of extreme responses (e.g., consecutive selection of the same option with obvious illogicality), those interrupted due to objective reasons such as emergency examinations for the child, and those with missing key variable information caused by the caregiver's voluntary withdrawal midway were all excluded. This ensured the quality of the questionnaires finally included in the data analysis.

### Data analysis

In this study, data processing and statistical analysis were carried out with the help of IBM SPSS Statistics 25.0 software. Firstly, descriptive statistical analysis was performed on the general demographic characteristics (including gender, age, educational level, etc.) of main caregivers of children with intracranial tumors after surgery and the scores of each dimension of the MCEM scale by using statistics such as mean, standard deviation, frequency and composition ratio. In the inferential statistical analysis stage, the research was carried out around the influencing factors of the empowerment ability of main caregivers. For binary variables (such as gender, whether suffering from chronic diseases, etc.), independent sample t-test was used to compare the differences in empowerment scores between different groups; for multi-category variables (such as educational level, occupation type, etc.), one-way analysis of variance (ANOVA) was used to analyze their impact on empowerment ability; Pearson or Spearman correlation analysis was used to study the strength and direction of the correlation between variables and empowerment ability scores; finally, multiple linear stepwise regression analysis was used to construct a prediction model to accurately screen out the key factors that have a significant impact on the empowerment ability of main caregivers. All statistical tests were conducted by two-tailed test, and the test level was set to α = 0.05.

## Results

### Characteristics of included caregivers

A total of 242 main caregivers were enrolled in this study. As presented in Table 1, the children had a mean age of 6.85 ± 2.04 years. Among the main caregivers, the majority were female (76.86%) and urban residents (76.03%), with a mean age of 37.14 ± 4.50 years. Additionally, 88.43% of the main caregivers were the children's parents.

**Table 1:** The characteristics of children with intracranial tumors and main caregivers.

Characteristic	Cases (%)	Empowerment	t/F	*p*
Child’ gender			2.184	0.103
Male	135 (55.79%)	159.05 ± 14.79		
Female	107 (44.21%)	162.18 ± 16.33		
Child’ age (y)			1.205	0.034
<5	45 (18.59%)	158.40 ± 15.41		
5–10	130 (53.72%)	160.66 ± 14.85		
11–18	67 (27.69%)	163.12 ± 15.66		
Place of residence			2.920	0.010
Rural area	58 (23.97%)	155.64 ± 16.05		
Urban area	184 (76.03%)	164.30 ± 15.23		
Medical payment expense method			1.557	0.174
Public medical insurance	190 (78.51%)	160.60 ± 15.13		
Commercial medical insurance	25 (10.33%)	161.24 ± 16.39		
Fully self-funded	21 (8.68%)	157.32 ± 15.01		
Other	6 (2.48%)	162.40 ± 15.75		
Caregiver's gender			1.255	0.038
Male	56 (23.14%)	156.24 ± 14.89		
Female	186 (76.86%)	161.02 ± 15.44		
Caregiver's age (y)			2.173	0.040
<40	137 (56.61%)	157.29 ± 16.34		
≥40	105 (43.39%)	163.85 ± 15.77		
Relationship with the child			1.794	0.202
Parent	214 (88.43%)	160.89 ± 15.47		
Other	28 (11.57%)	158.60 ± 14.34		
Educational level			1.004	0.021
Primary school or below	12 (4.96%)	155.26 ± 16.55		
Junior high school	48 (19.83%)	157.86 ± 15.13		
Senior high school or technical secondary school	113 (46.69%)	160.64 ± 14.87		
College or above	69 (28.51%)	164.29 ± 15.77		
Employment status			2.371	0.094
Employed	168 (69.42%)	161.24 ± 15.58		
Unemployed	74 (30.58%)	159.88 ± 16.04		
Monthly per capita income of family			1.336	0.019
<5,000 yuan	92 (38.02%)	157.60 ± 15.15		
≥5,000 yuan	150 (61.98%)	162.08 ± 14.85		
Having chronic diseases			2.004	0.072
No	174 (71.90%)	161.03 ± 16.44		
One	46 (19.01%)	158.50 ± 15.76		
Two or more	22 (9.09%)	157.83 ± 15.17		
Religious belief			1.276	0.101
No.	215 (88.84%)	160.44 ± 15.90		
Yes	27 (11.16%)	161.87 ± 15.29		
Average hours of daily care for the child			2.668	0.074
<12	72 (29.75%)	160.19 ± 14.60		
≥12	170 (70.25%)	161.20 ± 15.33		
Having other caregivers for the child			1.058	0.161
Yes	138 (57.02%)	160.57 ± 16.04		
No	104(42.98%)	159.01 ± 15.48		

### Empowerment scores

The average score of empowerment among main caregivers was 160.64 ± 15.07, indicating that there is still room for improvement. The scores of each dimension of Empowerment are presented in Table 2, among which the scores of “Personal Resources” and “Care Knowledge and Skills” are the lowest.

**Table 2:** Scores of each dimension of MCEM for main caregivers of children with intracranial tumors after surgery.

Dimension	Number of items	Average score for each item (*x*¯ ± *s*)
Personal Resources	7	2.71 ± 0.64
Subjectivity of Caregivers	6	3.32 ± 0.46
Care Beliefs	5	3.21 ± 0.58
Care Knowledge and Skills	3	2.84 ± 0.82
Concerns About the Surroundings	6	2.95 ± 0.77
Relationship with Care Recipients	6	3.65 ± 0.41
Benevolent Care	6	3.53 ± 0.45
Perception of Care's Role	6	3.09 ± 0.52
Expectations for Care Outcomes	6	3.51 ± 0.64

MCEM, main Caregivers’ empowerment measurement.

### Univariate analysis results

As shown in Table 1, statistically significant differences were observed in terms of the child's age, place of residence, caregiver's gender, caregiver's age, educational level, and monthly per capita income (all *p* < 0.05). In contrast, no statistically significant differences were found regarding the child's gender, medical expense payment method, relationship with the child, employment status, presence of chronic diseases, religious belief, average daily hours of care for the child, or presence of other caregivers for the child (all *p* > 0.05).

### Correlation analysis results

As indicated in Table 3, correlation analysis indicated that child's age (r = 0.581), place of residence (r = 0.546), caregiver's gender (r = 0.604), caregiver's age (r = 0.626), educational level (r = 0.615), and monthly per capita income (r = 0.586) were associated with the empowerment ability of children with intracranial tumors after surgery (all *p* < 0.05).

**Table 3:** Correlation analysis of empowerment ability and characteristics of main caregivers of children with intracranial tumors after surgery.

Variables	r	*p*
Child's gender	0.150	0.097
Child's age	0.581	0.023
Place of residence	0.546	0.041
Medical payment expense method	0.124	0.185
Caregiver's gender	0.604	0.031
Caregiver's age	0.626	0.017
Relationship with the child	0.134	0.095
Educational level	0.615	0.008
Employment status	0.108	0.077
Monthly per capita income	0.586	0.042
Having chronic diseases	0.114	0.105
Religious belief	0.201	0.069
Average hours of daily care for the child	0.131	0.144
Having other caregivers for the child	0.125	0.094

### Multivariate regression analysis results

As presented in Table 4, the multivariate linear regression analysis revealed that the child's age, place of residence, caregiver's gender, caregiver's age, educational level, and monthly per capita income were significant influencing factors of empowerment ability among main caregivers of children with intracranial tumors after surgery. The coefficient of determination (*R*^2^) for this regression model was 0.588, indicating that the selected variables could explain 58.8% of the variance in caregivers' empowerment ability. The overall model fit test yielded an *F*-value of 26.067 (*p* < 0.001), which confirms the statistical significance of the model's fitting effect.

**Table 4:** Multivariate linear regression analysis on the influencing factors of empowerment ability among the main caregivers of children with intracranial tumors after surgery.

Variables	Regression coefficient	Standard error	Standardized regression coefficient	t	*p*
Constant	156.03	4.005	–	38.631	0.002
Child's age	2.452	1.384	0.091	1.864	0.014
Place of residence	7.330	1.669	0.105	2.996	0.018
Caregiver's gender	5.407	1.247	0.268	2.703	0.044
Caregiver's age	4.975	1.025	0.101	1.294	0.025
Educational level	4.778	1.451	0.147	2.062	0.012
Monthly per capita income	5.960	1.003	0.099	1.245	0.007

*R*^2^ = 0.588, *F* = 26.067, *p* < 0.001.

## Discussion

### Overall level and improvement potential of empowerment among main caregivers of children with intracranial tumors after surgery

The results of this study indicate that the average empowerment score of main caregivers of children post-craniotomy is 160.64 ± 15.07, suggesting that their empowerment level is in the moderate range with substantial room for improvement. This finding aligns with conclusions from related research in the field of pediatric chronic disease care—due to the complexity of post-operative care for children with intracranial tumors (e.g., long-term rehabilitation needs, high risk of complications), caregivers often face multiple challenges in knowledge, skills, and resources, which restrict their ability to actively participate in care decision-making and integrate resources (13, 14). Compared with the empowerment levels of caregivers of children with chronic diseases such as leukemia or cerebral palsy, the scores of the study population are lower. This may be attributed to the higher professional requirements for post-craniotomy care and the greater difficulty in coordinating family care with medical interventions. These results imply that clinical practice needs to specifically strengthen systematic support for this group, addressing the shortcomings in their empowerment through targeted interventions to meet the demands of long-term post-operative care.

### Analysis of weak dimensions in empowerment—deficiencies in “Personal Resources” and “Care Knowledge and Skills”

This study identifies “Personal Resources” and “Care Knowledge and Skills” as the lowest-scoring dimensions of empowerment, a result with significant practical implications. The “Personal Resources” dimension reflects caregivers' perception and ability to mobilize available material, social, and psychological resources. Its low score may be related to the economic pressure and insufficient social support commonly faced by families of children with intracranial tumors—long-term treatment costs and care burdens may weaken caregivers' sense of control over resources, while access to resources is even more limited for rural or low-income families (15). The low score in “Care Knowledge and Skills” directly points to gaps in clinical training: post-craniotomy care involves specialized content such as neurological rehabilitation and complication prevention. Without systematic skill training (e.g., position management, medication guidance) provided by the medical team, caregivers are prone to a state of “passive care” due to knowledge deficits (16). This finding suggests that future interventions should prioritize resource linkage and skill training, supplementing these two dimensions through the establishment of a multidisciplinary support system (e.g., social worker involvement in resource integration, nurse-led practical skill training).

### Mechanism of the impact of child age on caregivers' empowerment

The results show that younger children are associated with lower empowerment among caregivers, a relationship that can be explained by the age-specific care needs of different developmental stages. Young children, especially infants and toddlers, have limited expressive abilities, making it more difficult to identify post-operative changes in their condition (e.g., inability to accurately describe symptoms such as headache or vomiting). Caregivers thus bear more complex observation and judgment tasks, and without targeted guidance, they may experience reduced confidence in active care due to anxiety or uncertainty (17, 18). Additionally, rehabilitation training for younger children (e.g., physical activity, cognitive stimulation) requires strategies more aligned with their developmental characteristics (19, 20). Caregivers without relevant knowledge often have diminished empowerment perceptions, as they struggle to implement effective interventions. Thus, clinical practice should offer more tailored guidance to families with younger children—including age-specific care manuals and development assessment-supported training—to enhance their capacity to address specific needs.

### Combined impact of Caregivers' individual characteristics and environmental factors on empowerment

This study finds that caregivers residing in rural areas, male caregivers, younger caregivers, and those with lower education or income levels exhibit significantly lower empowerment, highlighting the need to address the cumulative effects of these factors. From an environmental perspective, rural areas have poor access to medical resources (e.g., sparse distribution of rehabilitation institutions, insufficient professional care guidance), limiting caregivers' access to information and support, which may reduce their recognition of their own abilities (21). From an individual perspective, male caregivers, who traditionally play a less prominent role in main care within family structures, may have weaker empowerment perceptions due to inexperience or pressure from social role expectations (22). Meanwhile, lower education and income may indirectly weaken caregivers’ initiative in resource integration and decision-making participation by limiting information comprehension (e.g., interpreting medical documents) and increasing economic anxiety (23). This result is consistent with the theory of health disparities—socioeconomic status and geographic factors influence caregiver capacity building through unequal resource distribution (24). It suggests that interventions should balance environmental support (e.g., extending remote rehabilitation guidance to rural areas) and individual empowerment (e.g., developing simplified training for low-education caregivers) to break the cumulative effect of multiple disadvantageous factors (25).

### Implications for clinical nursing care

Synthesizing the results of this study, the core pathway to enhance the empowerment of main caregivers of children post-craniotomy can be summarized as “precision identification—stratified intervention—systematic support”. First, standardized assessment tools (e.g., the MCEM scale) should be used to identify high-risk groups (e.g., male caregivers in rural areas with low education caring for young children) to inform stratified interventions. Second, to address deficiencies in “Personal Resources”, a “medical-community-family” resource linkage network can be established—for example, social workers assisting in applying for medical assistance and connecting volunteer care support (26, 27). For weaknesses in “Care Knowledge and Skills”, a stepped training program (e.g., basic care → rehabilitation training → emergency response) should be designed, incorporating scenario simulations and video guidance to improve training effectiveness (28, 29). Finally, a family-centered multidisciplinary collaboration model should be promoted, integrating caregivers into medical decision-making processes (e.g., co-developing post-operative rehabilitation plans) to strengthen their sense of agency and, in turn, their empowerment (30–32). Future research should further explore the long-term effects of interventions, such as tracking the sustained impact of improved empowerment on children's rehabilitation outcomes and caregivers' well-being, to provide evidence for building a sustainable care support system.

## Limitations

Although this study systematically explored the current status and influencing factors of empowerment among main caregivers of children who have undergone craniotomy, it still has certain limitations that need to be addressed in subsequent research. First, this study adopted a single-center design, with all participants recruited from a single medical institution, which limits the geographical representativeness of the sample. Due to variations in medical resource allocation, caregiving cultures, and social support systems across different regions, the generalizability of the research findings may be restricted, making it difficult to fully reflect the empowerment characteristics of similar caregiver groups in diverse geographical areas. Additionally, the relatively small sample size of this study may lead to insufficient statistical power. Therefore, further verification through multi-center studies with larger sample sizes is warranted. Second, our study was limited to measurable factors like the child's age and caregivers' demographics. Yet empowerment, as a complex psychosocial concept, is likely shaped by additional unaccounted variables. These include the medical team’s communication approaches (e.g., use of family-centered decision-making), the family's collaborative care coordination, and the effectiveness of social support networks (e.g., practical care from relatives/friends, availability of community rehabilitation resources)—all of which may impact caregivers' empowerment perceptions through direct or indirect channels. Finally, this study is a cross-sectional survey, which can only reveal correlations between variables but cannot establish causal relationships or temporal effects. For example, there may be a dynamic interaction between caregivers' empowerment and caregiving burden, but the current research design cannot capture the process of this bidirectional influence. Future studies should employ a longitudinal follow-up design. This would involve multiple measurements to track changes in caregivers' empowerment over time, analyze its long-term correlations with children's rehabilitation outcomes and family function evolution, and provide more robust evidence for developing staged, individualized intervention strategies.

## Conclusion

In summary, the results of this study indicate that the empowerment ability of main caregivers of children who have undergone craniotomy is at a moderate level, with significant room for overall improvement. Among the dimensions, the weaknesses in “Personal Resources” and “Care Knowledge and Skills” are particularly prominent, serving as critical bottlenecks restricting their empowerment level. Further analysis indicates that caregivers' empowerment is jointly shaped by multiple factors. From the child's perspective, caregivers of younger children face greater empowerment challenges. From the caregiver and environmental perspective, significantly lower empowerment levels were observed among male caregivers, younger caregivers, those residing in rural areas, and individuals with lower education and income—reflecting the superimposed effects of individual characteristics and environmental factors. Our findings identify the core pain points and influencing mechanisms of empowerment among primary caregivers of children post-craniotomy, providing a critical basis for developing targeted clinical interventions. Future efforts should address weaknesses in “Personal Resources” and “Care Knowledge and Skills”. Interventions should specifically target high-risk groups, including rural male caregivers, those with low education and income, and caregivers of younger children. Constructing a multi-dimensional intervention system that balances resource support and capacity building may systematically improve caregiver empowerment, thereby enhancing family care quality for children's postoperative rehabilitation.

## Data Availability

The original contributions presented in the study are included in the article/Supplementary Material, further inquiries can be directed to the corresponding author.
